# The Moderating Role of Genetics: The Effect of Length of Hospitalization on Children’s Internalizing and Externalizing Behaviors

**DOI:** 10.3389/fpsyt.2015.00109

**Published:** 2015-08-17

**Authors:** Maya Benish-Weisman, Eitan Kerem, Ariel Knafo-Noam, Jay Belsky

**Affiliations:** ^1^Department of Counseling and Human Development, University of Haifa, Haifa, Israel; ^2^Division of Pediatrics, Hadassah Hospital, Jerusalem, Israel; ^3^Department of Psychology, The Hebrew University of Jerusalem, Jerusalem, Israel; ^4^Department of Human Ecology, University of California Davis, Davis, CA, USA

**Keywords:** hospitalization, length of hospitalization, emotional problems, behavioral problems, gene–environment interaction, DRD4, externalizing behavior, internalizing behavior

## Abstract

The study considered individual differences in children’s ability to adjust to hospitalization and found the length of hospitalization to be related to adaptive psychological functioning for some children. Applying the theoretical framework of three competing models of gene-X-environment interactions (diathesis–stress, differential susceptibility, and vantage sensitivity), the study examined the moderating effect of genetics (DRD4) on the relationship between the length of hospitalization and internalizing and externalizing problems. Mothers reported on children’s hospitalization background and conduct problems (externalizing) and emotional symptoms (internalizing), using subscales of the 25-item Strength and Difficulties Questionnaire (1). Data on both hospitalization and genetics were available for 65 children, 57% of whom were females, with an average age of 61.4 months (SD = 2.3). The study found length of hospitalization did not predict emotional and behavior problems *per se*, but the interaction with genetics was significant; the length of hospitalization was related to diminished levels of internalizing and externalizing problems only for children with the 7R allele (the sensitive variant). The vantage sensitivity model best accounted for how the length of hospitalization and genetics related to children’s internalizing and externalizing problems.

## Introduction

Hospitalization is a challenging experience for young children. It is frequently the sudden and unexpected result of disease or injury, leaving little time for preparation, and is often accompanied by physical discomfort or pain. Evidence reveals enduring effects on children’s internalizing and externalizing behaviors post-hospitalization (2, 3). Little is known, however, about longer-term effects extending beyond a year. The longer-term legacy of hospitalization is the focus of this report.

The effects of hospitalization, not surprisingly, may vary as a function of amount of time spent in the hospital. As it turns out, however, reported effects of hospitalization length on children’s emotional and behavioral functioning are mixed. Whereas some research indicates that longer periods of hospitalization predict more problematic functioning (4–6), other work suggests longer periods of hospitalization which are followed by recovery promote adaptive psychological functioning (7, 8). Especially notable are the results of a meta-analysis showing that hospitalization exceeding 3 days forecasts less negative behavioral change than do shorter stays of 2–3 days (9). Conceivably, children adjust over time to the new and unfamiliar environment of the hospital. In fact, it has been suggested that hospitalization affords the opportunity for emotional growth (10).

We hypothesize that the contrasting evidence on the effects of hospitalization length in the case of young children could be the result of some children simply being more susceptible to environmental effects than others. Therefore, our research tests the proposition that variation in the effects of hospitalization is likely to be a function not only of length of time spent in the hospital, but also of a child’s genetic make-up. The study design follows up a clinical sample at age five, a minimum of 2 years after their discharge, thereby affording us the opportunity to determine whether children with a certain genetic make-up are affected by their hospitalization more than others in terms of internalizing and externalizing behaviors. It is important to note that the correlational design of the study allows conclusions on relations, not causality. Accordingly, it is not clear whether hospitalization influences internalizing/externalizing problems or vice versa. Before providing specific design and measurement details, we turn to conceptual models of gene-X-environment (GXE) interaction and the role of genetics in shaping susceptibility to environmental influences.

### Conceptual models of gene-X-environment interaction

Most research on GXE interaction – the focus of the current inquiry – has been guided by the diathesis–stress model of environmental action (11). This theoretical perspective stipulates some individuals, due often to their personal characteristics – including their genetic make-up – are more likely to be negatively affected by contextual adversity. Certain genes which predispose individuals to succumb to adversity are regarded as “vulnerability genes” or “risk alleles” (12). Others not carrying these risk alleles are less likely to be negatively affected by contextual adversity and are considered “resilient.” In recent years, two alternative models of environmental action which can be applied to GXE have been advanced: differential susceptibility and vantage susceptibility.

#### Differential Susceptibility

The differential-susceptibility perspective presumes individual differences in developmental plasticity; that is, some individuals are more susceptible to the environmental regulation of their development than others (13–15). What distinguishes this theoretical model from the diathesis–stress perspective is the presumption that the very individuals whom the latter perspective presumes to be especially susceptible to the *negative* effects of contextual *adversity* are also disproportionately likely to *benefit* from a *supportive, enriched*, or benign environment. When this way of thinking is applied to the issue of effects of hospitalization, it suggests that for some young children – but not for others – longer periods of hospitalization will have positive effects on emotional and behavioral functioning, whereas shorter periods will have negative effects for some, but not for others. Thus, those carrying “plasticity genes” should, compared to those lacking them, show both more and less problematic functioning depending on whether, respectively, they spend less or more time in the hospital. As previous research suggests, a longer time in the hospital may allow children to adjust as they learn to use the various environmental resources, such as parents and medical staff, to overcome the hospitalization experience (16).

#### Vantage Sensitivity

An alternative to diathesis–stress and differential-susceptibility models of GXE interaction has recently been advanced (17). Essentially, it reflects only the “bright side” of differential-susceptibility thinking and, thus, is the exact opposite of the diathesis–stress model. Instead of conceptualizing some individuals as more susceptible to both positive and negative environmental influences (i.e., differential susceptibility) or to the negative effects of contextual adversity alone (i.e., diathesis–stress), “vantage sensitivity” presumes that some individuals are disproportionately likely to benefit from would-be positive environmental effects, while not being especially susceptible to the anticipated negative effects of contextual adversity. In other words, they may be predisposed to benefit from contextual support and enrichment – that is, to be developmentally sensitive to environmental advantage (18). Applied to the issue at hand, it predicts some children, due to their genetic make-up, will benefit more than others from longer hospitalizations, but children with a similar genetic make-up who experience short periods of hospitalization will not be more adversely affected than children carrying different genes.

The ultimate goal of the present inquiry is to determine which of the three theoretical models under consideration best accounts for how a particular gene moderates the effect of hospitalization length on young children’s emotional and behavioral functioning.

### DRD4

For many genes, the DNA sequence varies across individuals. These sequences are known as polymorphisms. We focus on a polymorphism in the third exon of the dopamine D4 receptor gene (DRD4-III). This polymorphism has two main variants that differ by the number of 48-base-pair tandem repeats in exon III: 7 present and 7 absence repeat allele. *DRD4* is involved in the limbic areas of the brain playing a role in emotional and cognitive functioning. More specifically, variations in this gene across individuals have been related to variations in attentional, motivational, and reward mechanisms (19). Studies have shown that DRD4 interacts with environmental stressors to predict externalizing behaviors among preschoolers concurrently (19, 20) and longitudinally (21). There is also some evidence of the importance of DRD4 to internalizing behaviors (20, 22). However, it has been shown that GXE interactions might be limited to specific populations (22) or not exist at all (23), stressing the importance of more studies.

Therefore, we focus on the presence vs. absence of the 7-repeat allele, first because of its relationship to variation in attentional and reward-related mechanisms which could conceivably influence how children respond to long vs. short hospitalizations. Second, we consider this putative “plasticity gene” because it has been found – perhaps more than any other polymorphism examined to date – to moderate environmental effects in a manner consistent with differential susceptibility (24).

## Materials and Methods

### Participants and procedure

Families in this study were drawn from the Longitudinal Israeli Study of Twins (LIST) examining genetic and socialization influences on development. All Jewish families identified by the Israeli Ministry of the Interior as having twins were contacted by mail close to the twins’ fifth birthday. Mothers were asked about the twins’ hospitalization history and behavior as well as additional information beyond the scope of this report (25). Children were asked to provide a genetic sample with parental permission. Data collection was approved by the Ethics Committees of the Herzog Hospital, Jerusalem, and the Hebrew University. Children who were hospitalized immediately after birth were excluded from the analysis, given their potential for developmental problems (26).

### Measures

Mothers reported on children’s conduct problems (externalizing) and emotional symptoms (internalizing) at age five, using subscales of the 25-item Strength and Difficulties Questionnaire (1), with five items comprising each subscale. The mothers rated responses on a scale from 0 = (*not true/seldom*) to 2 (*certainly true/very often*). Sample items are as follows: “Often fights with other youth or bullies them” (conduct problems); “Many worries or often seems worried” (emotional symptoms). Cronbach’s alpha was 0.62 for conduct problems and 0.64 for emotional symptoms.

#### Hospitalization History

*Hospitalization history* was assessed with questions to the parents about whether the children had been hospitalized, and if so, at what age and for how long. In addition, parents were asked to specify the reason for hospitalization.

#### DRD4-III Polymorphism

DNA was extracted by Master Pure kit (Epicentre, Madison WI) and PCR amplification was carried out. The exon III repeat region of the *DRD4* receptor was characterized by a PCR amplification procedure (using a Reddy Mix kit, AB gene, Surrey UK) with the following primers: F5′-TTCCTACCCTGCCCGCTCATGCTGCTGCTCATCTGG-3′; R5′-ACCACCACCGGCAGGACCCTCATGGCCTTGCGCTC-3′. We performed PCR reactions using 5 μl Master Mix (Thermo scientific), 2 μl primers (0.5 μM), 0.6 μl Mg/Cl2 (2.5 mM), 0.4 μl DMSO 5%, and 1 μl of water to total 9 μl volume; an additional 1 μl of genomic DNA was added to the mixture. All PCRs were employed on a Biometra T1 Thermocycler (Biometra, Güttingem, Germany).

## Results

### Preliminary analysis

Data on both hospitalization and genetics were available for 65 children (65% with 7R allele, 55% females; 35% 7-absent allele, 60% females), average age of 61.4 months (SD = 2.3). The extent of hospitalization ranged from 1 to 10 days (*M* = 4.08, SD = 2.67); 51.4% of the children were hospitalized between the ages of 1 and 12 months and 48.6% between the ages of 13 and 36 months. 28.8% were hospitalized for infections, 19.7% for surgical operations, 13.6% for diarrhea, and 9.1% for other problems; 28.8% did not answer this question. Children hospitalized more than once (*n* = 5) were excluded from the analyses.

The *DRD4-III* 7-repeat allele was not associated with the length of hospitalization (*r* = 0.07, n.s.). This rules out the possibility that this genetic characteristic might be directly related to the length of hospitalization and that any detected GXE interaction could be an artifact of gene–environment correlation involving this polymorphism. We found no relationship between the cause of hospitalization and the length of hospitalization [F(4) = 2.41, n.s.], enabling us to claim that the following results are hospitalization-length related and not hospitalization-reason related.

In addition, we found no main effect of sex or interaction of sex with DRD4 for conduct problems (*B* = −0.1, n.s., *B* = −38, n.s., respectively) or for emotional symptoms (*B* = 0.22, n.s., *B* = −0.36, n.s., respectively).

Similarly, there was no main effect of the age of hospitalization or the interaction of the age of hospitalization with DRD4 for conduct problems (*B* = −0.06, n.s., *B* = 0.03, n.s., respectively) or for emotional symptoms (*B* = −0.14, n.s., *B* = −0.47, n.s., respectively).

### Effect of length of hospitalization and genetics on problems

We examined the pathways between length of hospitalization and internalizing and externalizing behaviors with *DRD4* as a moderator in children aged five using Mplus (Muthén & Muthén, 1998–2007). Two models were tested, one for emotional (internalizing) problems and one for conduct (externalizing) problems.

#### Internalizing Behaviors

**Figure 1:**
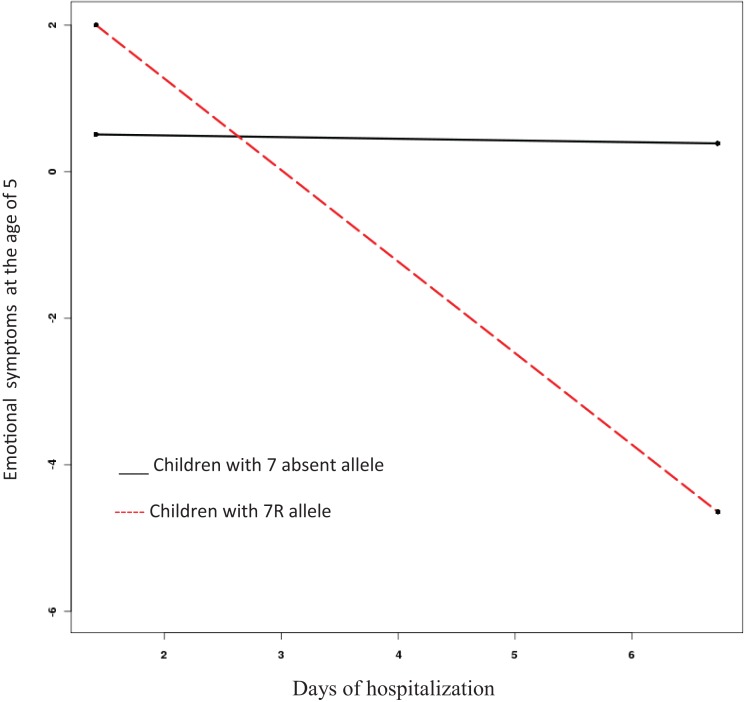
**Interaction effect of length of hospitalization and presence or absence of DRD4-III 7-repeat allele on emotional symptoms at age five**.

The model for emotional symptoms fit the data well (comparative fit index [CFI] = 1, root mean square error of approximation [RMSEA] = 0). The length of hospitalization was not associated with emotional symptoms (β = –0.14, SE = 0.14, Est./S.E. = −1.02, n.s.), although children with 7R allele experienced more emotional symptoms than those without it (β = 3.15, SE = 0.87, Est./S.E. = 3.63, *p* = 0.00). Importantly, the interaction between *DRD4-III* and length of hospitalization was significant (β = −2.88, SE = 0.92, Est./S.E. = −3.14, *p* = 0.002). Thus, the slope reflecting the relationship between length of hospitalization and emotional symptoms was calculated for each genetic sub-group and tested for significance (27). Whereas length of hospitalization proved unrelated to emotional symptoms in the case of children not carrying the 7R allele (β = −0.024, n.s.), for those carrying it, longer hospitalization predicted *fewer* emotional symptoms (β = 1.27, *p* = 0.01) (see Figure 1).

#### Externalizing Behaviors

**Figure 2:**
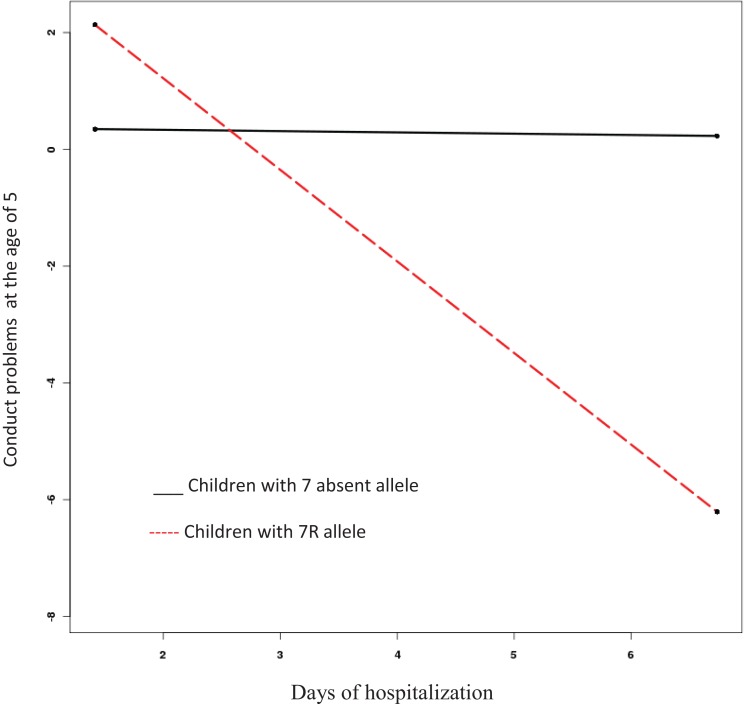
**Interaction effect of length of hospitalization and presence or absence of DRD4-III 7-repeat allele on conduct problems at age five**.

The model to predict conduct problems at age five fits the data well (CFI = 0.94, RMSEA = 0.06). As we found for emotional symptoms, length of hospitalization did not predict conduct problems at the age of five (β = −0.13, SE = 0.11, Est./S.E. = −1.14, n.s.). Nevertheless, children with 7-present allele had more conduct problems than those with 7-absent allele (β = 4.12, SE = 0.59, Est./S.E. = 6.96, *p* = 0.00). Furthermore, the interaction between *DRD4-III* and length of hospitalization was significant in predicting conduct problems among 5-year-olds (β = −4.19, SE = 0.59, Est./S.E. = −7.16, *p* = 0.00) (see Figure 2); specifically, for the children carrying the 7R-allele, more time in hospital predicted fewer conduct problems.

### Discounting alternative possibilities and choosing best fitting conceptual model

The analyses above are typical of studies of GXE interaction. Recently, however, there have been calls to conduct more rigorous tests to distinguish among conceptual models of GXE interaction (28–30).

This approach (29) addresses certain limitations in GXE interaction examination approaches. The first is the *visual inspection* limitation. Some previous studies have used different ranges of the independent variable (X, hospitalization length in the current study) to probe the interaction; others have not specified the range. If there are no standard criteria, different graphs can be sketched from the same data, leading to dissimilar or even contradictory conclusions. We followed the recommendation (29) to set extended interactions boundaries within the range of ± 1.5 SDs on X; this captured 93% of the sample and set a rigid criterion, greatly increasing the possibility that the results would be attributed to the appropriate GXE model.

The second limitation addressed in this approach (29) concerns *non-linearity*. Specifically, in cases where there is a non-linear predictor–outcome relationship, a diathesis–stress effect can be interpreted incorrectly as consistent with the differential susceptibility model. To rule out the possibility of non-linear relation between predictors and outcome, we tested X2 (the independent variable squared) and ZX2 (the moderator doubled the squared independent variable) in predicting emotional symptoms and conduct problems. (We refer to regression model Y = b0 + b1X + b2Z + b3XZ where X represents days of hospitalization and Z is the dichotomous moderator, *DRD4*.) We found that the relationship is indeed linear. We found no significant effect for emotional symptoms (β = −0.02, SE = 0.16, Est./S.E. = −1.4, n.s.; β = .−0.27, SE = 0.26, Est./S.E = −1.04, n.s.) or for conduct problems (β = −0.06, SE = 0.15, Est./S.E. = −0.39, n.s.; β = .−0.16, SE = 0.29, Est./S.E = −0.55, n.s.).

The third limitation (29) is the *lack of standard index for the quantification* of GXE models. To test which conceptual model fit our data, we calculated the *Proportion of Interaction* (PoI) index (29); this reflects the proportion of the total area of an interaction plot bounded by, in our case, ± 1.5 SDs on X that is uniquely attributable to the “better” side of the interaction. PoI values close to 1.0 support the vantage sensitivity model; PoI values close to 0.50 support the differential susceptibility model; and values closer to 0.00 support the diathesis–stress model. One of the main advantages of this index is that PoI is not directly affected by sample size as can be seen by the formula below. (B_3_ X_low_ + B_2_) and (B_3_ X_high_ + B_2_) are the simple slopes of Y (the dependent variable) on Z the moderator) at low and high X, respectively.

$$\text{Pol}={\left(\frac{B_{\text{3}}{\text{X}}_{\text{low}}\text{+}b_{\text{2}}}{B_{\text{3}}{\text{X}}_{\text{high}}\text{+}b_{\text{2}}}\right)}^{-1}$$

In our study, the PoI values were 0.98 and 0.82 for emotional symptoms and conduct problems, respectively. These results support the vantage sensitivity model over the differential-susceptibility or diathesis–stress models.

## Discussion

Hospitalization may have a major impact on children’s lives, including long-term effects on their development (2, 3). Although many previous studies have focused on short-term emotional and behavioral problems, our study followed once-hospitalized children for a much longer term. While all children were studied at age five, their period of hospitalization ranged from 2 to 4 years previously. Findings indicate that length of hospitalization in and of itself does not predict behavioral and emotional problems. Apparent effects – in this observational/correlational study – of length of hospitalization emerged only when a particular feature of children’s genetic make-up was taken into account.

Longer periods of hospitalization predicted fewer internalizing and externalizing behaviors at age five only for those children carrying the 7R allele. In fact, rather than detecting evidence consistent with either the diathesis–stress (11) or differential-susceptibility models (19–21) of GXE interaction, the results proved consistent with vantage sensitivity (29). That is, we did not find that the length of hospitalization was related to negative outcomes (diathesis–stress model) or that shorter periods of hospitalization were related to negative outcomes and longer ones to positive outcomes (differential sensitivity). This result challenges former findings that see hospitalization as only adverse (2, 3). Of course, most previous studies measured children’s reactions close in time to the hospitalization experience, while in our study, children’s behavior was measured 2 to 4 years after the hospitalization. It is possible that after a certain period of time, the overall experience of hospitalization was remembered as positive rather than negative for children carrying the 7R allele as we explain below.

The DRD4-III 7-repeat allele has repeatedly been shown to be associated with stronger relations between environmental variables and developmental outcomes, with this effect more pronounced in positive environments (17). We found the length of hospitalization was related to diminished levels of internalizing and externalizing problems among genetically sensitive children. A possible interpretation of the findings is that longer periods of hospitalization benefit sensitive children, at least in terms of their emotional-behavioral well-being. Arguably, longer hospitalization offers these children more opportunity to overcome the initial distress; they may gain comfort and a sense of control, resulting in a greater sense of security and, thus, develop fewer problems as the hospital routine becomes familiar and they adjust to a supportive environment that includes caring medical professionals and other hospital staff. They may also come to better understand the nature of their hospitalization experience. Graphs 1 and 2 reveal that the critical time for 7R allele children to adjust to hospitalization is the second or the third day. These results confirm former meta-analysis (9) showing hospitalization exceeding 3 days forecasts less negative behavioral change than do shorter stays of 2–3 days.

Parenting practices may need to be considered to gain further insight into why these particular findings emerged (31, 32). During hospitalization, young children often enjoy close parental care all day long and at night as well. Conceivably, then, longer periods of hospitalization may provide children with extra parental support and care, thereby promoting their emotional security and well-being. Future research should not only take into account variation in children’s susceptibility to environmental influences, but also the reasons for hospitalization and the nature of parental care during time in the hospital.

The findings are in line with some studies examining the effect of hospitalization on children’s emotional and behavioral problems (7, 8) but not with others (4–6). These mixed results may be accounted for by cultural differences in the prevalence of specific alleles across cultures or sub-cultures; that is, the sensitive allele might be frequent in some cultures but not in others. We suggest that in some cultures more children will be sensitive to environmental influence, resulting in different effects on children’s externalizing and internalizing problems post hospitalization (33). Most previous studies lack specification of participants’ cultural background; therefore, future studies should include this information. Another possible explanation is that the same genetic variation will have a different phenotype in different cultures (23). Future studies should expand the results to include more cultures, allowing us to determine whether they are universal or culture specific.

Despite the evident strengths of the current work, including the focus on genetic moderation of long-term “effects” of hospitalization and the effort to distinguish, via formal statistical criteria, the GXE model that best fits the data, this inquiry has limits. To begin with, the sample is not large, so future work would be well advised to use larger samples while seeking to replicate the findings reported here. An additional limitation is the exclusive focus on child behavior problems; it would be ideal if future work could examine a positive, not just a negative function (and/or its absence). Might the hospitalization experience have fostered the development of self-esteem, for example? Finally, the current design does not allow us to conclude causality. We have shown that length of hospitalization and genetic vulnerability may be related to the symptoms of the child at a later age, but causality should be examined in future studies that will test children before and after hospitalization.

As we note in this article, and as many others have similarly noted, hospitalization can be a major event for children. Whereas previous studies have highlighted the behavioral difficulties that follow hospitalization, this investigation sheds light on the brighter side of hospitalization by applying a candidate-gene and GXE approach to the inquiry.

## Conflict of Interest Statement

The authors declare that the research was conducted in the absence of any commercial or financial relationships that could be construed as a potential conflict of interest.
